# Human Neurospheres as Three-Dimensional Cellular Systems for Developmental Neurotoxicity Testing

**DOI:** 10.1289/ehp.0800207

**Published:** 2009-02-26

**Authors:** Michaela Moors, Thomas Dino Rockel, Josef Abel, Jason E. Cline, Kathrin Gassmann, Timm Schreiber, Janette Schuwald, Nicole Weinmann, Ellen Fritsche

**Affiliations:** Institut für Umweltmedizinische Forschung gGmbH an der Heinrich Heine-Universität, Toxicology, Düsseldorf, Germany

**Keywords:** apoptosis, differentiation, DNT, human neurospheres, mercury, migration, proliferation

## Abstract

**Background:**

Developmental neurotoxicity (DNT) of environmental chemicals is a serious threat to human health. Current DNT testing guidelines propose investigations in rodents, which require large numbers of animals. With regard to the “3 Rs” (reduction, replacement, and refinement) of animal testing and the European regulation of chemicals [Registration, Evaluation, and Authorisation of Chemicals (REACH)], alternative testing strategies are needed in order to refine and reduce animal experiments and allow faster and less expensive screening.

**Objectives:**

The goal of this study was to establish a three-dimensional test system for DNT screening based on human fetal brain cells.

**Methods:**

We established assays suitable for detecting disturbances in basic processes of brain development by employing human neural progenitor cells (hNPCs), which grow as neurospheres. Furthermore, we assessed effects of mercury and oxidative stress on these cells.

**Results:**

We found that human neurospheres imitate proliferation, differentiation, and migration *in vitro*. Exposure to the proapoptotic agent staurosporine further suggests that human neurospheres possess functioning apoptosis machinery. The developmental neurotoxicants methylmercury chloride and mercury chloride decreased migration distance and number of neuronal-like cells in differentiated hNPCs. Furthermore, hNPCs undergo caspase-independent apoptosis when exposed toward high amounts of oxidative stress.

**Conclusions:**

Human neurospheres are likely to imitate basic processes of brain development, and these processes can be modulated by developmental neurotoxicants. Thus, this three-dimensional cell system is a promising approach for DNT testing.

Developmental neurotoxicity (DNT) of environmental chemicals has been recognized worldwide as a serious threat to human health, and the resulting neurologic deficits negatively affect families and society (Goldman and Koduru 2000; Needleman et al. 2002). Current DNT testing guidelines (Organization for Economic Cooperation and Development 2007; U.S. Environmental Protection Agency 1998) propose investigations in rodents, mainly rats. Such a DNT *in vivo* testing strategy implies the use of 140 dams and 1,000 pups and is therefore extremely time- and cost-intensive (Lein et al. 2005). Relying solely on the existing guidelines to address current and anticipated future regulatory demands for DNT of the thousands of chemicals for which there are few to no DNT data would incur unacceptable costs in terms of animals and person-years (Lein et al. 2007). Therefore, according to the “3R principle” (reduction, replacement, and refinement) of Russel and Burch (1959), alternative testing strategies are needed to address animal welfare by refining and reducing animal experiments, and to create affordable, sensitive, and mechanism-based methods suitable for high- or medium-throughput screening (Collins et al. 2008). Furthermore, the inclusion of human-cell–based *in vitro* systems into an integrated DNT tiered testing approach has been recommended to circumvent species differences (Coecke et al. 2007).

To combine transatlantic strengths and avoid doubling of work, a partnership between the Johns Hopkins Center for Alternatives to Animal Testing (Developmental Neurotoxicity TestSmart program), the European Centre for the Validation of Alternative Methods, and the European Chemical Industry Council has been formed. This partnership follows the common goal of “incorporating *in vitro* alternative methods for DNT testing into inter national hazard and risk assessment strategies” (Coecke et al. 2007). Coecke et al. (2007) provided a comprehensive overview of the existing *in vitro* models and stated that, “although all the test systems described were not developed for regulatory purposes at this stage if they prove useful, we hope that this report will encourage their further development to render them amenable to high-throughput approaches.”

Therefore, the aim of this work was *a*) to introduce the cell biological characteristics of human neurospheres as a three-dimensional cell system approach for DNT testing; *b*) to demonstrate that neurospheres are likely to mirror basic processes of brain development, such as proliferation, differentiation, migration, and apoptosis; and *c*) to demonstrate that these processes can be modulated by developmental neurotoxicants.

## Materials and Methods

### Chemicals

We obtained methylmercury chloride (MeHgCl) from Riedel-de Haën (Seelze, Germany); all other substances were obtained from Sigma Aldrich (Munich, Germany), unless otherwise stated.

### Cell culture

Cryopreserved normal human neural progenitor cells (hNPCs; Lonza Verviers SPRL, Verviers, Belgium) were cultured at 37°C and 5% CO_2_ as a suspension culture in proliferation medium consisting of Dulbecco’s modified Eagle medium (DMEM) and Hams F12 (3:1) supplemented with B27 (Invitrogen GmbH, Karlsruhe, Germany), 20 ng/mL epidermal growth factor (EGF; Biosource, Karlsruhe, Germany), and 20 ng/mL recombinant human fibroblast growth factor (FGF; R&D Systems, Wiesbaden-Nordenstadt, Germany) (Moors et al. 2007). When spheres reached 0.7 mm in diameter, they were chopped up to passage 3 with a McIlwain tissue chopper. Differentiation was initiated by growth factor withdrawal in differentiation medium [DMEM and Hams F12 (3:1) supplemented with N2 (insulin, transferrin, sodium selenite, putrescine, and progesterone; Invitrogen)] and plated onto poly-d-lysine/laminin–coated chamber slides (BD Bioscience, Erembodegem, Belgium).

### Chemical exposure

We exposed cells to indirubin (10 μM) in proliferation medium (28 hr), and to cAMP (200 μM), MeHgCl (250 nM to 1 μM), mercury chloride (HgCl_2_; 500 nM to 10 μM, 48 hr) or staurosporine (0.1 and 1 μM), or hydrogen peroxide (H_2_O_2_; 0.1 and 1 mM) (24 hr) in differentiation medium. We chose concentration ranges of mercury according to Monnet-Tschudi et al. (1996), who found a concentration of 1 μM to be cytotoxic.

### Migration analyses

Migration analyses were performed as previously described (Moors et al. 2007). For living cell migration analyses, neurospheres were grown in the Focht Chamber System 2 (Bioptechs, Butler, PA, USA) under temperature- and CO_2_-controlled conditions. Images were acquired every 2 min by a Zeiss Axiovert 100 inverted microscope (Zeiss, Goettingen, Germany).

### Immunohistochemistry

Proliferating or differentiating spheres were fixed in 4% paraformaldehyde for 30 min. After washing spheres in phosphate-buffered saline (PBS), they were incubated in a 25% sucrose solution (wt/vol) overnight at 4°C. Afterward, spheres were transferred to tissue-freezing medium (Jung HistoService, Nussloch, Germany). Cryostat sections (10 μm) were prepared for immunohistochemistry. Antibodies for staining were nestin (1:150; BD Bioscience), glial fibrillary acidic protein (GFAP; 1:100, Sigma Aldrich), or β(III)tubulin (1:100; Sigma Aldrich).

### Immunocytochemistry/differentiation analyses

We performed immunocytochemistry as previously described (Fritsche et al. 2005; Moors et al. 2007). For quantification analyses, we used the Metamorph analysis software package (version 7.1.7.0; Universal Imaging Corp., West Chester, PA, USA). We determined the variation of protein expression by relating area of fluorescence signal to cell number in a region of interest within the migration area. Individual pixels were identified as “positive” if the fluorescence signal exceeded a determined color threshold [green, hue (H) 71–113, saturation (S) 10–255, intensity (I) 10–255; yellow/red, H 0–71, S 10–255, I 10–255)]. To determine the cell number, we selected images for positive 4′,6-diamidino-2-phenylindole (DAPI) staining (blue, H 152–180, S 10–255, I 10–255) and morphologic parameters (integrated morphology analysis: area, 10^4^> *n*> 10 ^7^; ellipsoid form factor, 0.1 > *n*> 1.8).

### Fluorescence-activated cell sorting (FACS)/cell cycle analyses

Proliferating neurospheres were grown in proliferation media with or without growth factors or exposed to indirubin. To obtain a single-cell suspension, neurospheres were washed once in PBS, incubated with Accutase (100%; PAA, Cölbe, Germany) at 37°C for 20 min, and then gently pipetted. The suspension was centrifuged (4°C, 1,400 × *g*, 5 min) and the pellet was resuspended in PBS containing 0.8% paraformaldehyde (Polyscience Inc., Eppelheim, Germany). Cells were fixed for 30 min at 4°C, centrifuged (4°C, 1,400 × *g*, 5 min), resuspended in PBS containing 0.15% saponin and 10 μg/mL RNase, and incubated for 30 min at 37°C. We then added 50 μg/mL propidium iodine 5 min before FACS analyses.

### Cell viability, apoptosis, and proliferation assays

We measured cell viability using the CellTiter-Blue assay (Promega, Mannheim, Germany) as previously described (Moors et al. 2007). The assay is based on measurements of the mitochondrial reductase activity by conversion of the substrate resazurin to the fluorescent product resorufin by mitochondrial reductases, which can be assessed in a fluorometer. The lactate dehydrogenase (LDH) assay (CytoTox-One; Promega) assesses cell death by measuring LDH that leaks out of dead cells into the media. We performed the assay according to the manufacturer’s instructions. Briefly, supernatants of treated cells were collected and incubated with an equal amount of CytoTox-One reagent for 4 hr before the detection of fluorescence (excitation, 540 nm; emission, 590 nm). Caspase-3/-7 activities were measured with the Apo-One Kit (Promega) according to the manufacturer’s instructions. Briefly, cells were lysed and caspase activity was assessed by measuring the cleavage of a caspase-3/-7–specific fluorescent substrate (Z-DEVD-R110) with a fluorometer (excitation, 488 nm; emission, 538 nm).

For proliferation assays, spheres were cultured in proliferation medium supplemented with or without 20 or 100 ng/mL EGF in 96-well plates. We assessed cell viability as a measure for cell number using the CellTiter-Blue assay at different time points. Because the dye caused no acute cytotoxicity, spheres were washed with medium after fluorescence reading and then the same spheres were monitored over a period of 2 weeks. For determination of sphere size, we gauged sphere diameter optically with an object micrometer. We counted the number of cells/sphere after trypsination (0.25% trypsin; Invitrogen) for 2 min.

### TUNEL assay

For terminal deoxy-nucleotidyl transferase 2′-deoxyuridine 5′-triphosphate (dUTP) nick end labeling (TUNEL) assays, we used fluorescein-coupled dUTP and the terminal transferase kit from Roche Diagnostics (Mannheim, Germany) to label DNA strand breaks; nuclei were counter-stained with Hoechst 33258 (Invitrogen). Plated neurospheres were exposed to staurosporine (1 μM) or H_2_O_2_ (1 mM) after 48 hr of differentiation. After another 12 and 24 hr, cells were fixed with 4% paraformaldehyde, washed twice with PBS, covered with reaction mixture (2.5 mM CoCl_2_, 5 μM fluorescein coupled dUTP, 5,000 U/mL terminal-transferase, 2 μg/mL Hoechst, 0.1% triton in 1× terminal transferase buffer), and incubated at 37°C for 1 hr. Slides were then washed with PBS three times and mounted with PBS/glycerol (1:1). Stained cells were analyzed with a fluorescence microscope.

### Statistics

We used analysis of variance combined with the Bonferroni post hoc test for multifactor analyses (time and concentration effects), and the Student’s *t*-test for two-group comparisons (treatment vs. control; two time points). The significance value was set at *p* < 0.05. To describe the associations between independent variables (diameter/cell number; diameter/fluorescence), we fitted curves up to the third degree. We used *R*^2^ as a measure of goodness of fit.

## Results

Human neurospheres grow floating freely in defined medium without addition of serum [see Supplemental Material, Figure 1A (available at http://www.ehponline.org/members/2009/0800207/suppl.pdf)]. Upon withdrawal of growth factor, cells migrate radially out of the sphere onto a poly-d-lysine/laminin matrix, thereby forming a migration area [see Supplemental Material, Figure 1B and the video (available at http://www.ehponline.org/members/2009/0800207/suppl.pdf)]. Each cell leaves the sphere edge in a 90° angle and travels away in a straight line. Moreover, cells move toward and away from each other.

To evaluate reproducibility and stability of neurosphere migration, we assessed dependence of migration speed on neurosphere size. Therefore, the distance between the sphere edge and the farthest outgrown cells was measured 24 hr after plating, dependent on different sphere diameters. Supplemental Material, Figure 1C (available at http://www.ehponline.org/members/2009/0800207/suppl.pdf) shows that spheres with a diameter between 0.2 and 0.7 mm wander approximately 0.48 mm within 24 hr (e.g., 0.2 mm diameter, 0.48 ± 0.06 mm; 0.7 mm diameter, 0.48 ± 0.09 mm; mean ± SD), demonstrating that migration speed is independent of sphere size. Moreover, cells from different donors (0.3-mm-diameter spheres) also did not vary significantly in migration speed over 24 hr [see Supplemental Material, Figure 1D (available at http://www.ehponline.org/members/2009/0800207/suppl.pdf)].

**Figure 1:**
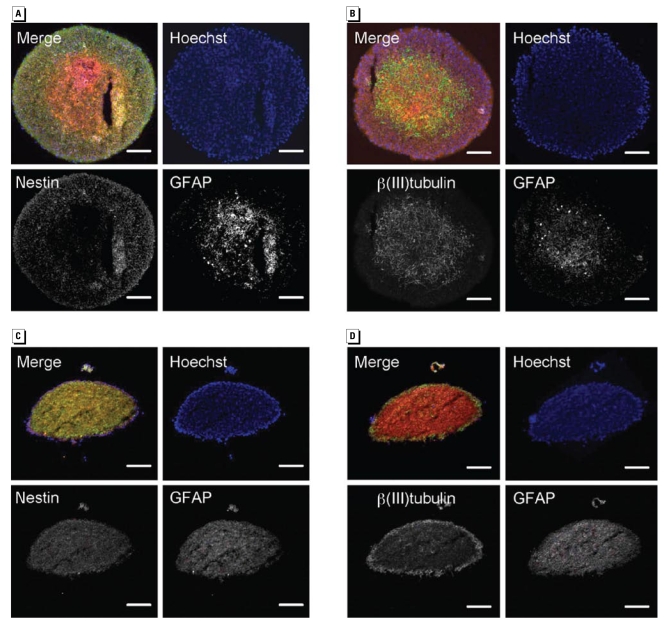
Cellular composition of human neurospheres shown in cryostat sections (10 μm) of proliferating (*A* and *B*) and differentiating (8 days after plating; *C* and *D*) neurospheres (representatives of five spheres for each developmental stage). Nuclei are stained in blue with Hoechst; nestin and β(III)tubulin are stained in green; and GFAP is stained in red. Individual antibody stainings are shown as contrast images. Bars = 100 μm.

Next, we analyzed the cellular composition of neurospheres. We sliced 10-μm cryostat sections of proliferating neurospheres and examined expressions of *a*) nestin, a marker protein for neural stem cells; *b*) β(III)tubulin, which stains neurons; or *c* ) GFAP for glial cells. Immunocytochemical analyses revealed nestin-positive (^+^) cells were located mainly in the sphere periphery, whereas β(III)tubulin^+^ and GFAP^+^ cells resided in the sphere center (Figure 1A,B). This pattern disappeared after spheres were plated for differentiation. After 8 days of differentiation, β(III)tubulin^+^ cells were located at the edge of the sphere, whereas nestin^+^ and GFAP^+^ cells were homogenously distributed throughout the sphere (Figure 1C,D).

**Figure 2:**
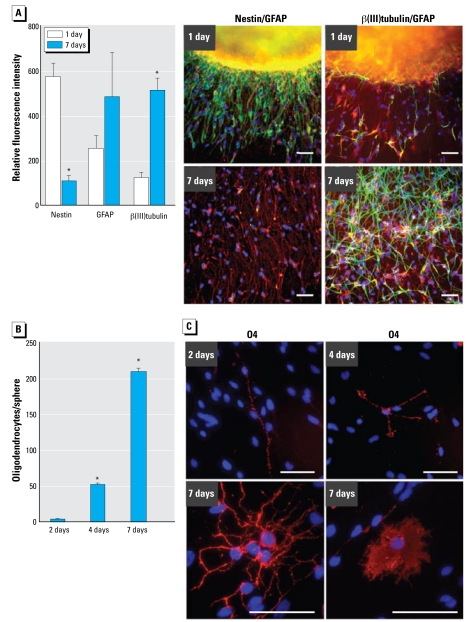
Cellular composition of the neurosphere migration area. (*A*) Quantification of nestin^+^, GFAP^+^, and β(III)tubulin^+^ pixels after 1 and 7 days of differentiation (mean ± SD) and representative photo graphs [left: nestin (green), GFAP (red); right: β(III)tubulin (green), GFAP (red)]. Ten individual spheres were included in each calculation. (*B*) Quantification of migrated O4^+^ cells, counted manually after 2, 4, and 7 days of differentiation in six individual spheres (mean ± SD). (*C* ) Morphology of O4^+^ cells at different time points. Bars = 100 μm. *p = 0.05.

In addition to the sphere itself, we investigated the cellular composition of the migration area after 24 hr and 7 days of migration. Twenty-four hours after plating, nearly all migrated cells seemed to express nestin, showing that immature cells migrate out of the sphere. Furthermore, β(III)tubulin^+^ and GFAP^+^ cells were also located in the migration area. In contrast, 7 days after plating almost all cells lost nestin expression and became β(III) tubulin^+^ or GFAP^+^ (Figure 2A). Quantification of the number of pixels in the respective images revealed a 5.5-fold reduction in the number of nestin^+^ pixels/nuclei after 6 more days of differentiation (570.9 ± 64 to 103.2 ± 29 pixels/nuclei; mean ± SD), whereas in the same time period the number of β(III)tubulin^+^ and GFAP^+^ pixels increased 4.7- and 1.9-fold, respectively [β(III)tubulin, from 118.7 ± 27.4 to 509.6 ± 55 pixels/nuclei; GFAP, from 250.8 ± 56 to 480.7 ± 198 pixels/nuclei; Figure 2A]. Furthermore, the immunocytochemical staining for β(III)tubulin suggests that neuronal cells may form connections and thus build neuronal networks (Figure 2A).

Another cell type emerging from neural precursor cells are O4^+^ oligodendrocytes. They form within the neurosphere (Fritsche et al. 2005) and migrate out of the sphere over time. After 2, 4, and 7 days of differentiation, 3 ± 0.2, 52 ± 1, and 210 ± 5 O4^+^ cells (mean ± SD), respectively, were located in the migration area (Figure 2B). They also changed morphology over time. Although after 48 hr most O4^+^ cells were bipolar, we found more branching after 4 days; after 7 days of differentiation, multipolar and membrane sheet-forming cells were prominent (Figure 2C).

Next, we developed assays that identify changes in cell proliferation, differentiation, migration, and apoptosis by applying model chemicals, which are known to interfere with normal brain development (Grandjean and Landrigan 2006).

**Figure 3:**
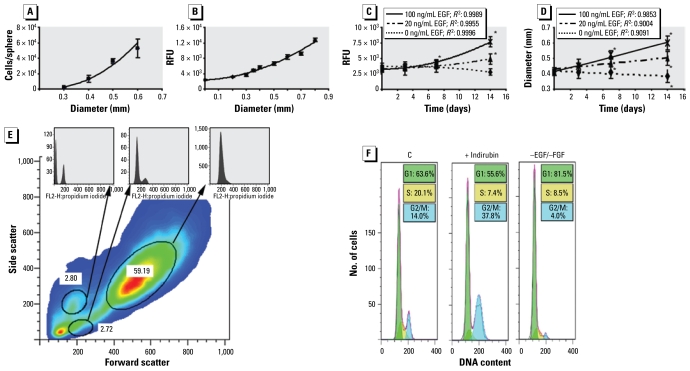
Assessment of neurosphere proliferation. RFU, relative fluorescence units. (*A*) Correlation between sphere diameter and number of cells/sphere (*R*^2^ = 0.9926). (*B*) Correlation between sphere size and metabolic activity; reductase activity was measured with the CellTiter-Blue assay (*R*^2^ = 0.983). (*C*) Measurement of sphere proliferation by assessing metabolic activity repetitively over time. (*D*) Assessment of proliferation in the same spheres shown in (*C*) by measuring sphere diameter over time. Results in (*C* ) and (*D*) are typical representatives of three independent experiments at each time point shown as mean ± SD of 3–6 individual spheres. (*E*) FACS analysis of dissociated, fixed, and propidium iodine–stained neurospheres. The circled regions depict subpopulations; one contained proliferating cells (see second DNA histogram). (*F* ) DNA content histograms of the proliferating cell population. The control histogram corresponds to cells cultured with EGF and FGF. A G_2_/M or G_1_ arrest was induced with indirubin (28 hr) or by withdrawal growth factor (96 hr). *p = 0.05.

Cell proliferation in a neurosphere can be determined by counting the number of cells per dissociated sphere or by measuring the increase in sphere diameter over time. Figure 3A shows that there was a very good association between these two parameters (e.g., 2.6 × 10^3^ and 5.3 × 10^4^ cells for spheres 0.3 and 0.6 mm in diameter, respectively). We verified this observation and made the method suitable for high-throughput analyses by measuring viability of neurospheres dependent on sphere diameter with the CellTiter-Blue assay. Figure 3B demonstrates that viability of spheres correlates well with their sizes (e.g., spheres 0.3 and 0.6 mm in diameter had 4 × 10^3^ and 8 × 10^3^ relative fluorescence units, respectively). Growth of neurospheres in the absence or presence of 20 or 100 ng/mL EGF caused a 1.5 ± 0.4-fold or 2.4 ± 0.3-fold induction in viability (mean ± SD), respectively over 14 days, although the same spheres gained 0.08 ± 0.03 or 0.2 ± 0.04 mm in diameter, respectively, during this time (Figure 3C,D). We observed no differences in proliferation between spheres grown in EGF in the presence or absence of FGF (data not shown). Cultivation without growth factors as a negative control did not change size or viability. Thus, this assay can assess sphere proliferation.

We verified these data by FACS analyses for DNA content using propidium iodine staining. Among all stained cells, 97.75% showed only a G_0_/G_1_ peak (Figure 3F), whereas we found a typical cell cycle distribution for proliferating cells in only 2.72% of the population. About 35% of these were in G_2_/M or S phase (Figure 3F, control), suggesting fast cell-cycling behavior. Indirubin, a G_2_/M blocking agent that blocks signaling of cyclin-dependent kinases (Hoessel et al. 1999), increased the cell fraction in G_2_/M phase from 14% to 37.8% (Figure 3F), whereas withdrawal of growth factors caused G_1_ arrest.

**Figure 4:**
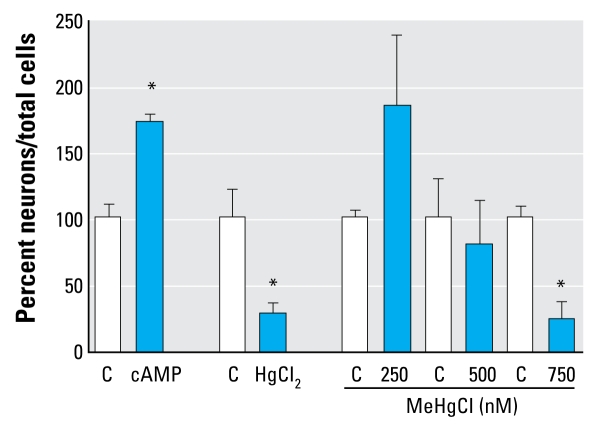
Chemicals disturb neurosphere differentiation. C, control. β(III)tubulin^+^ cells/total nuclei were counted after exposing differentiating neurospheres to chemicals for 48 hr. Data are mean ± SD of at least three independent experiments (3–5 spheres per experiment).

To investigate effects of chemicals on differentiation, we exposed neurospheres to different Hg compounds. Immunocytochemical analyses after 48 hr revealed that migration areas of control cells consist of 10% β(III)tubulin^+^ cells and 90% GFAP^+^ cells (Moors et al. 2007). MeHgCl (500 and 750 nM) reduced the amount of β(III)tubulin^+^ cells to 8 ± 0.17% (mean ± SD) and 2.3 ± 0.57%, respectively. Exposure to 4 μM HgCl_2_ decreased the number of β(III)tubulin^+^ cells to 4.7 ± 2.3%. In contrast, cAMP increased the formation of β(III)tubulin^+^ cells to 165.4 ± 9% of control cells (Figure 4).

**Figure 5:**
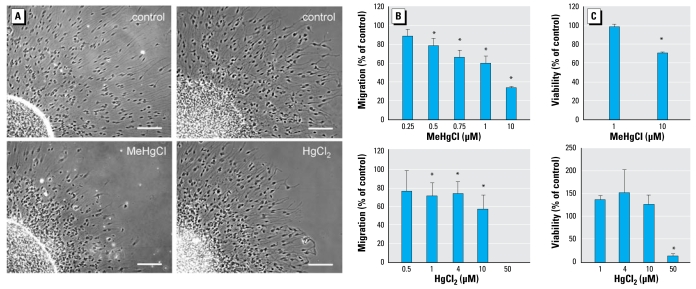
Mercury inhibits neurosphere migration. Phase-contrast images (*A*) and the respective quantifications (*B*) of cell migration (migration distance measured with an object micrometer from the edge of the sphere to the farthest outgrowth). In (*A*), bars = 100 μm. (*C*) Cell viability as assessed with the CellTiter-Blue assay. In (*B*) and (*C*), data are mean ± SD of at least three independent experiments (3–5 spheres/experiment). *p< 0.05.

Next, we investigated the effects of Hg on cell migration with the neurosphere migration assay (Moors et al. 2007). Exposure to MeHgCl (500 nM) caused an inhibition of cell migration to 78.7% ± 7% of control values, which was further reduced by higher MeHgCl concentrations. HgCl_2_ (4 μM) also reduced cell migration to 73.6 ± 13% of the controls (Figure 5A, B). Notably, cell migration was significantly affected by noncytotoxic Hg concentrations (Figure 5C).

**Figure 6:**
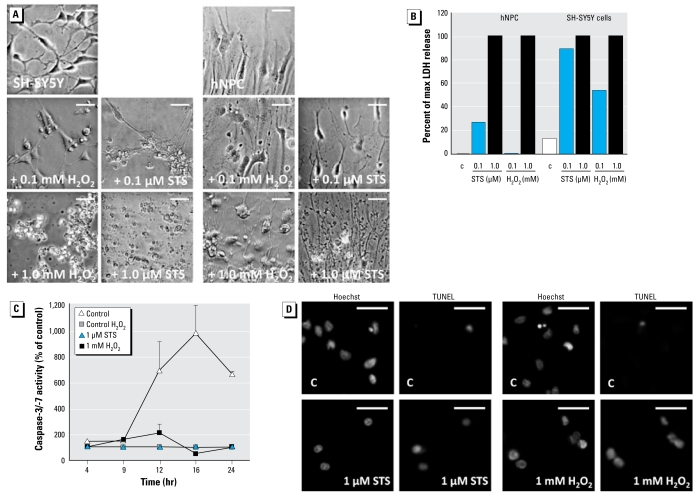
STS but not H_2_O_2_ induces caspase-dependent apoptosis. Abbreviations: C, control; max, maximum; STS, staurosporine. (*A*) Phase-contrast images and (*B*) corresponding LDH release of SH-SY5Y cells and hNPC after 24 hr of STS or H_2_O_2_ treatment. (*C*) Kinetic analyses of caspase-3/7 activity after STS or H_2_O_2_ treatment; values are typical representatives of two independent experiments (three spheres/experiment). (*D*) Cells showing positive TUNEL staining after STS or H_2_O_2_ exposure; nuclei are visualized with Hoechst. In (*A*) and (*D*), images are representative of two independent experiments; bars = 30 μm.

To determine whether human neurospheres can be stimulated to undergo apoptosis, we exposed them to staurosporine, a potent inducer of the intrinsic apoptotic pathway via cytochrome c release followed by activation of the caspase cascade (Slee et al. 2000), or H_2_O_2_, a direct reactive oxygen species (ROS) donor, for 24 hr. LDH measurements of neurosphere supernatants indicate that staurosporine and H_2_O_2_ induce cell death in a concentration-dependent manner. However, the human neuroblastoma cell line SH-SY5Y (ATCC, Wesel, Germany) is more susceptible to staurosporine- and H_2_O_2_-induced LDH release than are the spheres, as indicated by a higher LDH release at lower concentrations, which we confirmed using phase-contrast microscopic images (Figure 6A,B). To explore whether staurosporine (1 μM) or H_2_O_2_ (1 mM) induced cell death via apoptosis, we performed TUNEL assays. Although the basal apoptosis rate of hNPCs after 3 days of differentiation was approximately 1% (data not shown), both treatments induced TUNEL-positive cells, showing that apoptosis is involved in staurosporine-induced and H_2_O_2_-induced cell death (Figure 6D). However, measurements of effector caspase-3/-7 activities indicate that staurosporine-induced cell death is caspase dependent, whereas H_2_O_2_-triggered cell death is caspase independent (Figure 6C).

## Discussion

In humans, DNT results in learning deficits and mental retardation (Hass 2006; Schettler 2001). Furthermore, various clinical disorders (e.g., schizophrenia, autism) are results of interference with normal brain development, and their etiologies are suspected to also imply environmental components (Rice and Barone 2000). To prevent harm, it is crucial to understand DNT potentials of chemicals, and thus testing is necessary. Therefore, we established and characterized this three-dimensional human neurosphere system that imitates the basic processes of brain development—proliferation, differentiation, and migration [Figure 2; see also Supplemental Material, Figures 1 and 2 (available at http://www.ehponline.org/members/2009/0800207/suppl.pdf)].

Individual spheres in single wells of a 96-well plate proliferated over time, and FACS analyses of propidium iodine–stained neurosphere single-cell suspensions revealed that approximately 2.72% of sphere cells went through S-phase of the cell cycle, confirming their proliferative capacity (Figure 3E,F). This is in agreement with Reynold and Rietze (2005), who found 2.4% of human neurosphere cells capable of proliferation as assessed by a single-cell clonogenic assay.

To illuminate the inside of the “black-box” neurosphere, we immunocytochemically stained proliferating spheres. Microscopic analyses illustrate a zonal distribution of nestin^+^ hNPCs in the periphery and later GFAP^+^ and β(III)tubulin^+^ astrocytes and neurons in the center of the sphere (Figure 1). These findings are similar to data reported for murine neurospheres (Campos et al. 2004) and might be caused by a growth factor gradient from the sphere periphery to its inside. One could speculate that this zonal distribution resembles an “outside-in” brain structure, with nestin^+^ cells representing the proliferative zone of the brain, which is in proximity to the growth-factor–containing liquor of the ventricles, and the GFAP^+^ and β(III)tubulin^+^ cells in the center of the sphere resembling superficial regions of the cortex (Campos et al. 2004). Whether the growth factor gradient is in fact responsible for zonal dissemination within a neurosphere is a subject for future investigations.

Growth factor withdrawal and presence of a poly-d-lysine/laminin matrix initiate cellular migration out of the sphere (Moors et al. 2007). Observation of initial migration over 24 hr by real-time phase-contrast microscopy illustrates that radial as well as tangential migration happens during this time [see Supplemental Material, Figure 2 (available at http://www.ehponline.org/members/2009/0800207/suppl.pdf)]. The cues causing cells to connect, disconnect, and move forward, backward, and even tangentially *in vitro* are so far unknown. Directed migration *in vivo* is motivated by chemical gradients of, for example, Netrin1/UNC6, semaphorins, or the reelin/dab1 pathway (Hatten 2002). Although some of these gene products, such as different semaphorins, are expressed in our neurospheres (Moors M, Fritsche E, unpublished data), whether such attractants or repellants are responsible for the directed migration we observe *in vitro* has to be further investigated. Nevertheless, the distance that differentiating hNPCs migrate over time is highly robust and reproducible. Migration speed is independent of neurosphere size and does not differ between three independent donors tested so far [gestational weeks 16–19; see Supplemental Material, Figure 1 (available at http://www.ehponline.org/members/2009/0800207/suppl.pdf)]. A faster migration speed (1 mm/24 hr) was reported for neurospheres that were prepared from post-natal brain cortices of premature infants (gestational weeks 23–25; Flanagan et al. 2006). This difference might be due to distinct culture conditions or ages of individuals.

During cellular outgrowth, hNPCs differentiate into GFAP^+^, O4^+^, and β(III)tubulin^+^ glial- and neuronal-like cells while losing nestin staining (Figure 2A). The ratio of approximately 10% neuronal and 90% glial cells that we counted after 2 days of differentiation (Moors et al. 2007) resembles the physiologic distribution of brain cells in humans (Baumann and Pham-Dinh 2001). Furthermore, we found O4^+^ oligodendrocyte precursor cells in the migration area that increase in number and degree of morphologic maturation over time (Figure 2) (Baumann and Pham-Dinh 2001). These differentiation results point to culture maturation.

For the development of *in vitro* assays that identify chemicals with DNT potential, we employed Hg, which is a human developmental neurotoxicant (Grandjean and Landrigan 2006). Prenatal Hg poisoning causes developmental delays, mental retardation, and adverse effects on memory and motor skills in children (Sanfeliu et al. 2003; Schettler 2001). Neuropathologic examinations revealed microcephaly and global brain disorganization resulting from disturbances in cell migration and division (Clarkson 2002; Schettler 2001). Moreover, postmortem brains had a decreased number of nerve cells (Castoldi et al. 2001; Choi 1989). We mimicked these effects *in vitro* by treating neurospheres with organic Hg and inorganic Hg, which were identified in human brain sections (Clarkson 2002). Hg decreased the migration distance (Figure 5A,B) and increased the glial cell/neuron ratio (Figure 4). We observed these effects at noncytotoxic concentrations, pointing to a target-cell–specific effect (Figure 5C). How do these findings correspond to Hg exposures in humans? From an Hg poisoning incident in Iraq, a lowest observed adverse effect level (LOAEL) for brain MeHg content in intoxicated mothers was calculated to be 800 ng/g, a level that caused neurologic symptoms in children (Clarkson 1993). Considering the measurements of Lewandowski et al. (2003), who determined cellular *in vitro* concentrations relative to corresponding medium Hg concentrations, this LOAEL is equivalent to an *in vitro* medium concentration of approximately 266 nM. MeHg accumulation in the fetus is higher than in adult organs, implying that the LOAEL is under- rather than overestimated. *In vitro* exposure of rodent neural stem cells to HgCl_2_ (7–18 μM) or MeHg (2.5–5 nM) also resulted in reduced neuronal differentiation (Cedrola et al. 2003; Tamm et al. 2006). Although the sensitivity toward inorganic Hg was similar in human compared with mouse spheres (Cedrola et al. 2003), rodent stem cells treated for 7 days were more sensitive toward organic Hg than were human cultures treated for 2 days (Tamm et al. 2006). Although both mercuric compounds exert adverse effects by binding to sulfhydryl groups of proteins (Clarkson 1972), one further mode of action of organic Hg is the induction of oxidative stress (Sarafian and Verity 1991; Yee and Choi 1994). Antioxidant defenses are low in human embryonic brains and evolve during development (Buonocore et al. 2001). Furthermore, there might also be species differences in defense capacities (Knobloch et al. 2008). Thus, the differences between our and previously published results for MeHg could be due to the age of cultures (stem vs. fetal cells), species differences, or varying exposure times.

In contrast to Hg, cAMP, a well-described compound for inducing neuronal differentiation (Deng et al. 2001), caused an increased number of β(III)tubulin^+^ cells in differentiated hNPCs (Figure 4A), demonstrating the dynamic ability of the cell system.

Deregulation of apoptosis results in develop mental brain pathology or neuro-degenerative diseases (Rodier 1995). Furthermore, oxidative stress induces apoptosis in many different cells types. Therefore, we attempted to trigger ROS-induced programmed cell death in hNPC cells. Although staurosporine induced caspase-dependent apoptosis, 1 mM H_2_O_2_ induced TUNEL^+^ apoptotic cells without caspase-3/-7 activation, indicating that neurospheres undergo caspase-independent apoptosis (Figure 6). This is in concert with studies in primary rat cerebellar granule cells, which also responded with caspase-independent apoptosis to H_2_O_2_ (Dare et al. 2001). Furthermore, comparison of LDH activity of human hNPCs with the human neuroblastoma SH-SY5Y tumor cell line suggests that hNPCs are less sensitive toward oxidative stress than are SH-SY5Y tumor cells. These data also support observations that cancer cells are more susceptible to various stressors than are normal cells (Aykin-Burns et al. 2008; Hileman et al. 2004). Besides inducing apoptosis, preconditioning of mouse NPCs with a low concentration of H_2_O_2_ (5 μM) is cytoprotective (Sharma et al. 2008). Whether this is also true for hNPCs or even tumor cell lines needs to be investigated.

In summary, we have shown that *a*) proliferation, migration, differentiation, and apoptosis of human neurospheres can be quantified; *b*) *in vivo* effects of the developmental neurotoxicant Hg are imitated *in vitro*; and *c*) the methods applied are suitable for medium-throughput screening. Thus, our three-dimensional neurospheres offer a new, human, system-based method for DNT hazard identification. However, their applicability is limited to basic processes of brain development, because they do not resemble complex higher brain structure development such as formation of cortical layers. Moreover, they are limited in their potential to perform drug metabolism, as is fetal tissue *in vivo*. For including “maternal metabolism” in the *in vitro* system, strategies such as incubation with S9 mixes or hepatocyte coculture have to be established.

In the future, more chemicals known to cause DNT will be tested for their potential to interfere with human neurosphere performance to develop this method into a validation process and make it applicable for testing needs.
